# Highly sensitive label-free biosensor: graphene/CaF_2_ multilayer for gas, cancer, virus, and diabetes detection with enhanced quality factor and figure of merit

**DOI:** 10.1038/s41598-023-43480-5

**Published:** 2023-09-27

**Authors:** Behnam Jafari, Elnaz Gholizadeh, Bahram Jafari, Moheimen Zhoulideh, Ehsan Adibnia, Mahdi Ghafariasl, Mohammad Noori, Saeed Golmohammadi

**Affiliations:** 1 Faculty of Electrical and Computer Engineering, University of Tabriz, Tabriz, 5166616471 Iran; 2 Department of Electrical and Computer Engineering, University of British Columbia, Vancouver, Canada; 3 Department of Pharmacology, I.M. Sechenov First Moscow State Medical University (Sechenov university), Moscow, Russia; 4 Faculty of Electrical and Computer Engineering, University of Sistan and Baluchestan (USB), Zahedan, Iran; 5 Department of Physics and Astronomy, University of Georgia, Athens, GA 30602 USA; 6 Electrical Engineering Department, Technical and Engineering Faculty, University of Bonab, Bonab, East Azerbaijan Iran

**Keywords:** Nanophotonics and plasmonics, Optical sensors, Biomedical engineering, Breast cancer, Bionanoelectronics, Biosensors, Nanostructures, Optics and photonics, Graphene, Nanobiotechnology, Nanomedicine, Nanoscale devices, Nanoscience and technology, Nanoscale materials, Metamaterials, Breast cancer, Skin cancer, Biomaterials - cells, Biomedical materials, Engineering, Electrical and electronic engineering, Terahertz optics, Applied physics, Biological physics, Electronics, photonics and device physics, Bionanoelectronics, Biosensors, Nanofabrication and nanopatterning, Nanoparticles, Nanostructures

## Abstract

One of the primary goals for the researchers is to create a high-quality sensor with a simple structure because of the urgent requirement to identify biomolecules at low concentrations to diagnose diseases and detect hazardous chemicals for health early on. Recently graphene has attracted much interest in the field of improved biosensors. Meanwhile, graphene with new materials such as CaF_2_ has been widely used to improve the applications of graphene-based sensors. Using the fantastic features of the graphene/CaF_2_ multilayer, this article proposes an improvement sensor in the sensitivity (S), the figure of merit (FOM), and the quality factor (Q). The proposed sensor is based on the five-layers graphene/dielectric grating integrated with a Fabry–Perot cavity. By tuning graphene chemical potential (µ_c_), due to the semi-metal features of graphene, the surface plasmon resonance (SPR) waves excited at the graphene/dielectric boundaries. Due to the vertical polarization of the source to the gratings and the symmetry of the electric field, both corners of the grating act as electric dipoles, and this causes the propagation of plasmonic waves on the graphene surface to propagate towards each other. Finally, it causes Fabry–Perot (FP) interference on the surface of graphene in the proposed structure's active medium (the area where the sample is located). In this article, using the inherent nature of FP interference and its S to the environment's refractive index (RI), by changing a minimal amount in the RI of the sample, the resonance wavelength (interferometer order) shifts sharply. The proposed design can detect and sense some cancers, such as Adrenal Gland Cancer, Blood Cancer, Breast Cancer I, Breast Cancer II, Cervical Cancer, and skin cancer precisely. By optimizing the structure, we can achieve an S as high as 9000 nm/RIU and a FOM of about 52.14 for the first resonance order (M_1_). Likewise, the remarkable S of 38,000 nm/RIU and the FOM of 81 have been obtained for the second mode (M_2_). In addition, the proposed label-free SPR sensor can detect changes in the concentration of various materials, including gases and biomolecules, hemoglobin, breast cancer, diabetes, leukemia, and most alloys, with an accuracy of 0.001. The proposed sensor can sense urine concentration with a maximum S of 8500 nm/RIU and cancers with high S in the 6000 nm/RIU range to 7000 nm/RIU. Also, four viruses, such as M13 bacteriophage, HIV type one, Herpes simplex type 1, and influenza, have been investigated, showing Maximum S (for second resonance mode of λ_R_(M_2_) of 8000 nm/RIU (λ_R_(M_2_) = 11.2 µm), 12,000 nm/RIU (λ_R_(M_2_) = 10.73 µm), 38,000 nm/RIU (λ_R_(M_2_) = 11.78 µm), and 12,000 nm/RIU (λ_R_(M_2_) = 10.6 µm), respectively, and the obtained S for first resonance mode (λ_R_(M_1_)) for mentioned viruses are 4740 nm/RIU (λ_R_(M_1_) = 8.7 µm), 8010 nm/RIU (λ_R_(M_1_) = 8.44 µm), 8100 nm/RIU (λ_R_(M_1_) = 10.15 µm), and 9000 (λ_R_(M_1_) = 8.36 µm), respectively.

## Introduction

Recently, Optical biosensors have been the most efficient since they can detect biomolecules directly and instantly^1,2^. Because of this quick real-time detection, optical biosensors are also often used in medical applications^2^, environmental applications^3^, industrial applications^4^, etc. There are two primary approaches for using optical biosensors to find or sense biomolecules. Fluorescence is the foundation of the first detection approach, while label-free detection is the second. Of these two techniques, fluorescence-based detection employs biomolecular tags to find them. Although the Fluorescence approach is accurate, labeling all the samples makes it hard to use. On the other hand, this approach is expensive. Therefore, the second method has been widely used^5^.

The label-free detection of biomolecules can be realized by the excitation of SPR in waveguides, photonic crystal fibers, and Bragg gratings^6–8^. In contrast with the first approach, label-free techniques measure an inherent property of the query itself (e.g., mass and dielectric property), thereby avoiding modifying interactors^9^. The successes of sensing technologies are determined mainly by their S, resolution, and detection limit. Dynamic range, real-time monitoring, multiplexing and HT capability, widespread applicability, and data handling are other key determining factors^10,11^.

In protein microarray experiments, signals can be detected by label-based or label-free strategies. Both approaches have their merits and demerits. Label-based detection methods require labeling query molecules with fluorescent dyes, radioisotopes, or epitope tags. Label-based detection is widely used in protein microarrays due to the expected availability of reagents and simple instrument requirements. However, these labeling strategies often alter the query molecule's surface characteristics and biological activities. Moreover, the labeling procedure is laborious and lengthy, limiting the number and types of query molecules that can be studied^12^.

Meanwhile, in many cancer situations, SPR sensors are helpful for drug discovery^13^. SPR technology detection in biosensors can also limit drug-serum interactions^14^. Using localized surface plasmon-based biosensors, which can detect biomolecules, facilitates the diagnosis of Alzheimer's disease^15^.

In recent years, with the emergence of graphene and its applications in the electronics and photonics industry, such as optical modulators^16–19^, photodetectors^20,21^, sensors^22,23^, and Optical Tweezers^24–26^, graphene has been in the center of researchers' attention. This single-layer graphite allotrope known as graphene has unique optical and electrical features such as high carrier mobility, electrostatic doping, ultra-wide absorption spectrum^27^, easy fabrication while being compatible with well-established silicon technology^28^, and ultrafast charge carrier dynamics, which is originated from its 2D nature^29^. Nowadays, due to graphene's extraordinary optical and electrical characteristics, graphene-based SPR sensors are generating interest. It has been reported that graphene-based SPR sensors are suitable for biological applications with great features. Additionally, a graphene layer on the sensing medium results in superior conductivity and stable biomolecule absorption. The large carbon-based rings in biomolecules are the primary cause of a biomolecule's absorption by a graphene sheet^30^.

Graphene-based SPR sensors employ the shift in the graphene plasmon resonance wavelength to identify slight variations in the RI of the medium close to the graphene-based structure. Graphene-based sensors have made considerable advances in terms of sensing speed and structuring. Likewise, it can absorb bio and polar molecules well due to its high surface-to-volume ratio. Therefore, using graphene, electromagnetic waves could be coupled to the bound collective charge oscillations, forming localized SPR (LSPR).

Recently, prominent studies involving graphene grating structures have been reported. For instance, a graphene plasmonic grating ultrasensitive tunable terahertz sensor, which consists of SiO_2_ and graphene, has been reported^31^. The simulation results demonstrate multi-resonant modes suitable for ultrasensitive refractive index sensing. Also, a graphene-gold grating in the near-IR region has been proposed^32^, which performs well for detecting biomolecules and harmful gases for health. Absorption spectra of this structure indicate two resonance modes with S as high as 1100 nm/RIU and 1180 nm/RIU.

This paper proposes a structure and a novel method based on propagating plasmonic waves interferometer on graphene/CaF_2_ multilayer for detecting and sensing changes in the RI of samples (and thus the concentration) of gases, biomolecules, urine, and generally all materials with a refractive index between 1.000 and 1.800. Also, some cancers such as Adrenal Gland Cancer, Blood Cancer, Breast Cancer I, Breast Cancer II, Cervical Cancer, and skin cancer have been investigated precisely. This structure consists of five graphene layers separated by CaF_2_ layers and covers the grating structure's steps that form strong SPs in the mid-IR region. CaF_2_ has been used as a substrate material for graphene not only to increase the graphene plasmonic waves quality (due to zero imaginary part of permittivity in the wavelength range of interest) but also due to the high dielectric constant and its breakdown voltage, it causes to shift graphene chemical potential with very low gate voltage. Moreover, due to an ingenious method in which a polarized light source causes both edges of gratings to act as an electrical dipole, the excited plasmonic waves propagate toward each other and form an FP interference. This FP interferometer makes the primary mechanism of the proposed biosensor. Since FP interference is highly sensitive to its medium RI, the proposed design shows significant improvement in S, FOM, and Q. Additionally, research has been done on the potential sensing properties of various gas alloys and biomolecules. The proposed sensor can sense urine concentration with a maximum S of 8500 nm/RIU and cancers with high S in the 6000 nm/RIU range to 7000 nm/RIU. Also, four viruses, such as M13 bacteriophage, HIV type one, Herpes simplex type 1, and influenza, have been investigated, showing a Maximum S of 8000, 12,000, 38,000, and 12,000 nm/RIU, respectively.

## Structure and methods

**Figure 1:**
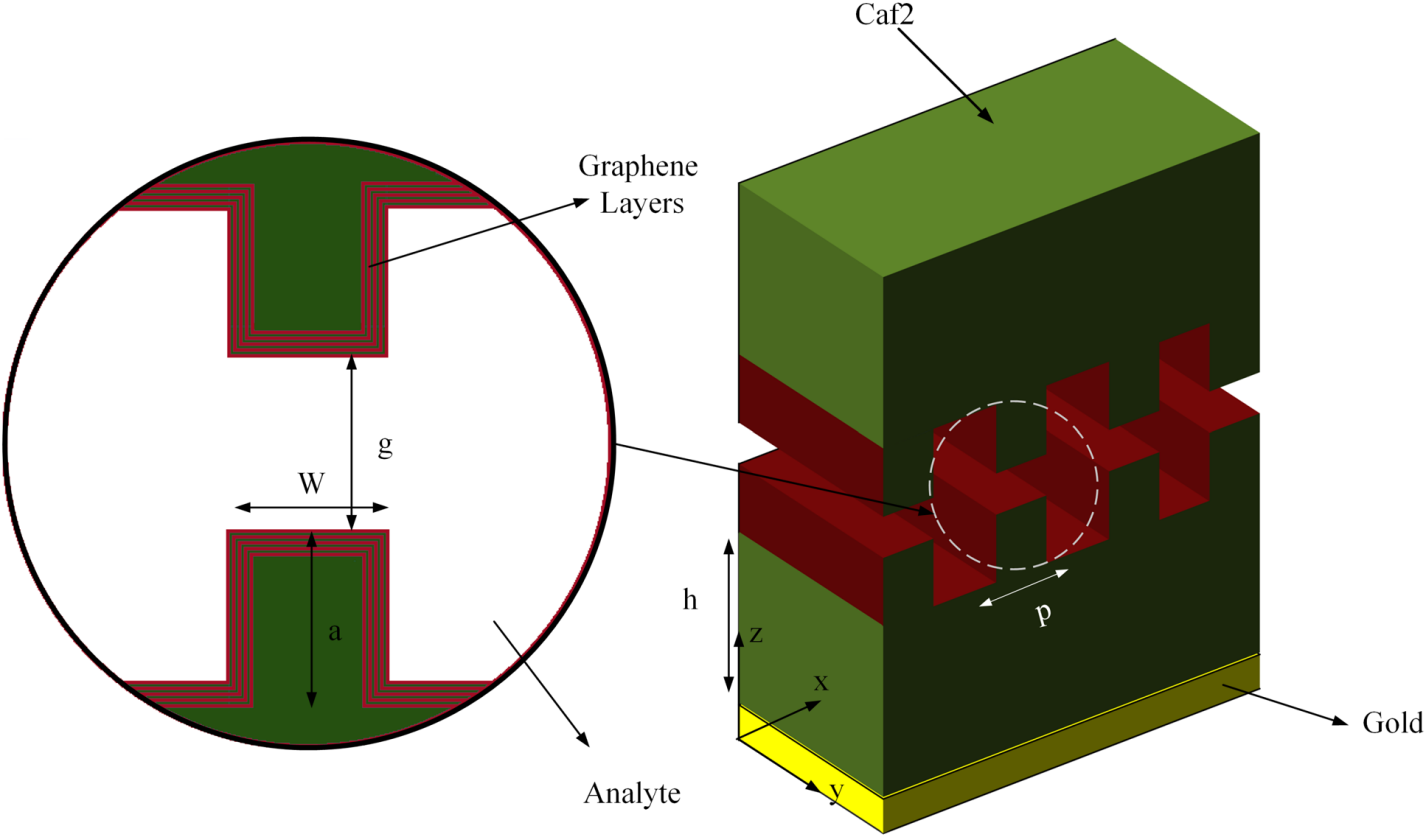
3D (right) and cross-section (left) view of the proposed structure for the biosensor application.

A 3D view of the proposed sensor and its cross-section is shown in Fig. 1. A grating-based structure is used to couple the incident wave with graphene's SPP waves. Furthermore, to increase plasmon wave quality, five graphene layers are enclosed in 4 layers of 2-nm thickness CaF_2_ layers and cover the surface of gratings, which results in high electric field confinement. In addition, these gratings are created from CaF_2,_ which is on the top of the gold substrate and serves as the gate voltage for adjusting the Fermi level of the graphene. In addition, to obtain realistic results, we rounded the grating corners in all simulations.

This paper uses graphene as a 2D sheet with its surface conductivity. The surface conductivity of graphene can be separated into interband conductivity, which is the first term of Eq. (3), and intraband conductivity^26^.

1 $$\begin{gathered} \sigma \left( {\omega ,\mu_{c} ,\Gamma ,T} \right) = \sigma_{{{\text{inter}}}} + \sigma_{{{\text{intra}}}} = \frac{{2e^{2} k_{B} T}}{{\pi \hbar^{2} }}.\frac{j}{{\omega + j\tau^{ - 1} }}.\ln \left[ {2\cosh \left( {\frac{{E_{f} }}{{2k_{B} T}}} \right)} \right] \hfill \\ + \frac{{e^{2} }}{4\hbar }\left[ {H\left( {{\omega \mathord{\left/ {\vphantom {\omega 2}} \right. \kern-0pt} 2}} \right) + j\frac{2\omega }{\pi }\int\limits_{0}^{\infty } {\frac{{H\left( {{{\omega^{\prime}} \mathord{\left/ {\vphantom {{\omega^{\prime}} 2}} \right. \kern-0pt} 2}} \right) - H\left( {{\omega \mathord{\left/ {\vphantom {\omega 2}} \right. \kern-0pt} 2}} \right)}}{{\omega^{2} - \omega^{{\prime}{^{2} }} }}d\omega^{\prime}} } \right] \hfill \\ H(\omega ) = {{\sinh ({{\hbar \omega } \mathord{\left/ {\vphantom {{\hbar \omega } {k_{B} T}}} \right. \kern-0pt} {k_{B} T}})} \mathord{\left/ {\vphantom {{\sinh ({{\hbar \omega } \mathord{\left/ {\vphantom {{\hbar \omega } {k_{B} T}}} \right. \kern-0pt} {k_{B} T}})} {\left[ {\cosh ({{E_{f} } \mathord{\left/ {\vphantom {{E_{f} } {k_{B} T}}} \right. \kern-0pt} {k_{B} T}}) + \cosh ({{\hbar \omega } \mathord{\left/ {\vphantom {{\hbar \omega } {k_{B} T}}} \right. \kern-0pt} {k_{B} T}})} \right]}}} \right. \kern-0pt} {\left[ {\cosh ({{E_{f} } \mathord{\left/ {\vphantom {{E_{f} } {k_{B} T}}} \right. \kern-0pt} {k_{B} T}}) + \cosh ({{\hbar \omega } \mathord{\left/ {\vphantom {{\hbar \omega } {k_{B} T}}} \right. \kern-0pt} {k_{B} T}})} \right]}} \hfill \\ \end{gathered}$$

In Eq. (1), E_f_ is the Fermi energy, $\hbar$ is the reduced Plank constant, k_B_ is the Boltzmann constant, T = 300 K is the temperature, e is the elementary charge, ω is the angular frequency, and τ is the relaxation time, respectively. Here the intrinsic relaxation time is taken to be $\tau = \mu .\mu_{c} /e.\nu_{f}^{2}$ .

**Figure 2:**
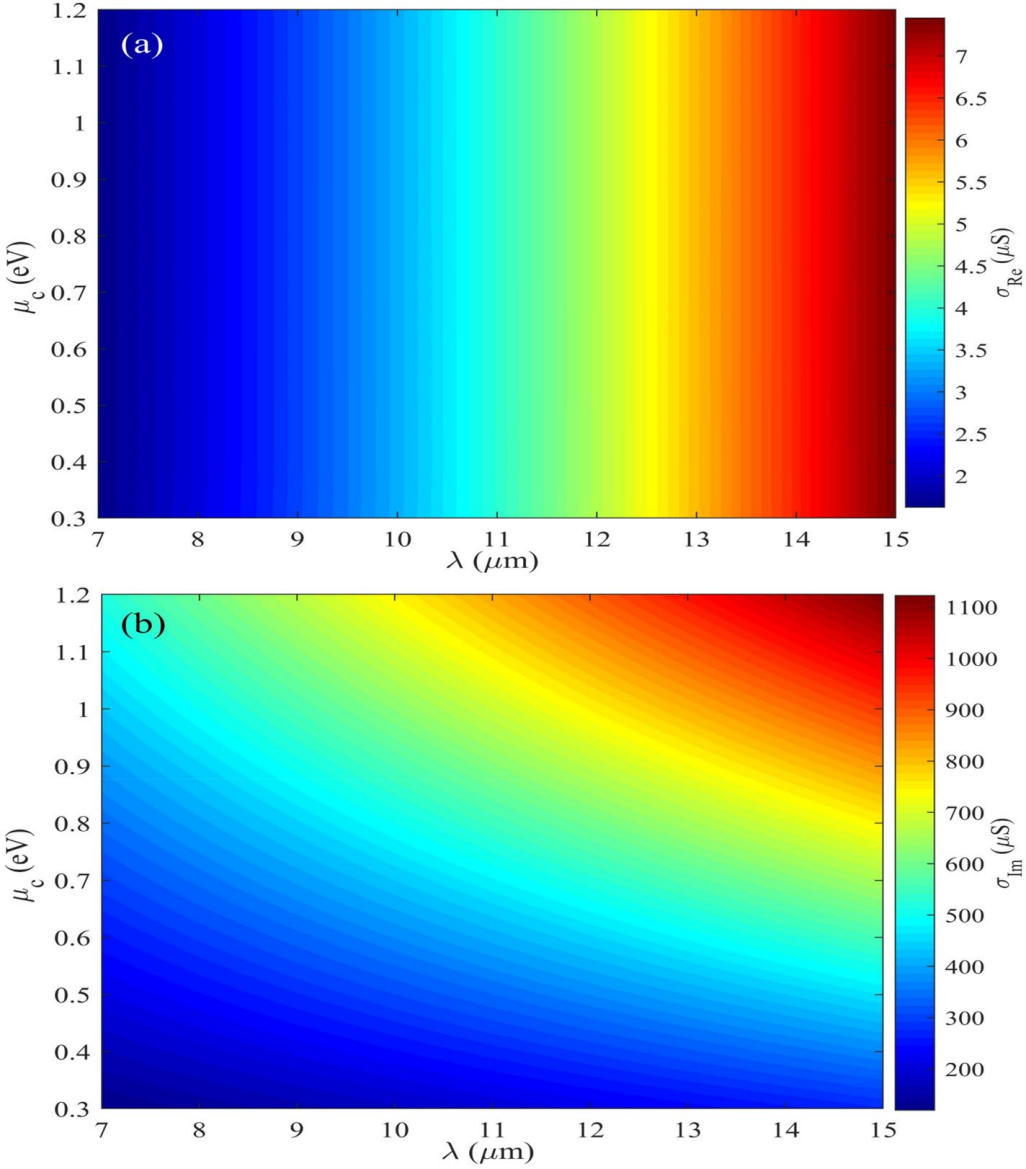
Real (**a**) and imaginary (**b**) part of graphene surface conductivity.

The scattering rate is obtained by $\Gamma = 1/2\tau$, µ is the mobility of the graphene on the Caf_2_ layer and is taken 1 m^2^/Vs.,^33,34^. Figure 2a,b illustrate real and imaginary parts of graphene surface conductivity derived from Eq. (1).

The parameters shown in Fig. 1 and used in Eq. (1) are defined in Table. 1.

**Table 1:** The parameters used in the proposed structure geometry and its modeling.

Parameter	Description	Value
p	Graphene grating's pitch	200 nm
w	Gap cavity width in the x direction	100 nm
a	grating's (gap) depth	200 nm
h	Depth of CaF2	550 nm
µ	Graphene on SiO2 mobility	1 m^2^ V^−1^ s^−1^
V_F_	Fermi velocity	10^6^ m s^−1^
n_0_	Residual charge carrier density	8.1 × 10^11^ cm^−2^

Most previous spp-based sensors consist of a prism substrate, a dielectric layer, novel metal film, liquid, and plasmon, which can only be excited by TM polarization (Kretschmann configuration) and have a large footprint after that, grating-based graphene sensors reached interests of researchers. Grating couplers have not been used in SPR sensors as widely as prism couplers. However, their compatibility with mass production and the prism's absence make grating-coupled SPR attractive for fabricating low-cost, compact-sensing devices.

Given the fact that the structure is based on wavelength absorption and the changes in the RI can be obtained as Eq. (1), the S of the proposed sensor is calculated as follows:

2 $$S = \frac{{\Delta \lambda_{\max } }}{\Delta n}$$

where Δλ_max_ is the change in the maximum wavelength of the absorption curve for a slight shift (Δn) in the RI of the material we want to measure, the figure of the merit (FOM) for the proposed sensor is calculated as Eq. (4):

3 $$FOM = \frac{S}{FWHM}$$

In addition, the Q for the proposed sensor can be calculated by:

4 $$Q = \frac{\lambda }{FWHM}$$

And the FWHM is the full-width pick at half the maximum absorption curve.

The infrared (IR) area is the optimum spectral range for biomolecule sensing since it doesn't result in photodamage. An optical sensor's detection accuracy will gradually increase if operated in the spectrum's infrared (IR) region since bio samples absorb considerably more in this region than in the visible area, which may also be connected to penetration depth towards the sample side.

A suitable substrate material should have good transparency, low refractive index (RI) discrepancy by varying the temperature, a big transmission window, and a high laser damage threshold in the Mid-IR spectral range. Like calcium fluoride (CaF_2_), fluoride glass can meet the qualities mentioned earlier among other substrate glass materials for applications in the IR region. CaF_2_ with a low RI value can also provide superior error rejection (mainly angular) during mechanical setup. Consequently, CaF_2_ might be a suitable substrate for SPR-based IR applications^35^. Therefore, recently it has been shown that CaF_2_-based SPR sensor provides much better S than conventional dielectrics such as SiO_2_.

**Figure 3:**
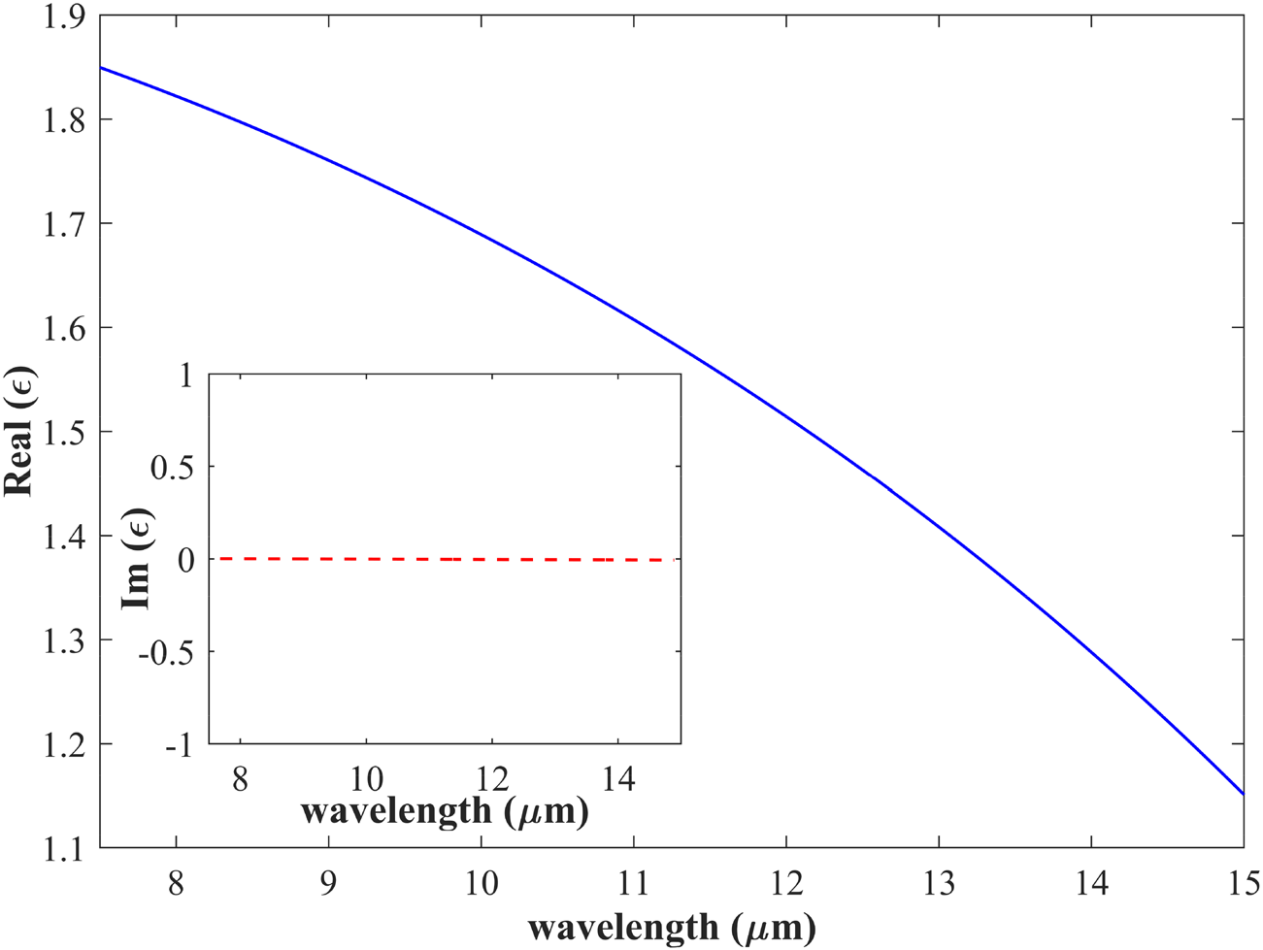
The real part of permittivity of the CaF_2_. _I_nset shows the imaginary part of permittivity (inset). As can be seen, the imaginary part of permittivity is zero, making the CaF_2_ a proper dielectric and spacer material for graphene in the wavelength of interest.

The approached experimental data of the permittivity of CaF_2_ are shown in Fig. 3. The dispersion formula of CaF_2_ is attained from the empirical Sellmeier approximation Eq. (6)^36^:

6 $$\varepsilon_{r,CaF2} = 1 + \frac{{0.5675888\lambda^{2} }}{{\lambda^{2} - 0.050263605^{2} }} + \frac{{0.4710914\lambda^{2} }}{{\lambda^{2} - 0.1003909^{2} }} + \frac{{3.8484723\lambda^{2} }}{{\lambda^{2} - 34.649040^{2} }}$$

where ε_r_ is the dielectric function, and λ is the wavelength in μm.

As shown in Fig. 3 and its inset, the imaginary part of permittivity is zero, making the CaF_2_ a proper dielectric and spacer material for graphene in the wavelength of interest. Also, the mentioned features of CaF_2_ make it a very suitable substrate for graphene, preventing secondary effects and increasing graphene improvement.

The optical analysis uses the FDTD method, and a normal incident with a linearly polarized source is employed to illuminate the structure. Its E-field is polarized perpendicularly to the gratings (in the x-direction). It is worth mentioning that the incident light that polarized parallel to the grating’s direction (in the y-direction) cannot activate the plasmon resonance because the structure entirely reflects it. The boundary conditions in the z-directions are Perfectly Matched Layers (PMLs), while the x and y directions are periodic.

## Results and discussions

One way to determine an analyte's concentration or perform a biophysical analysis is to evaluate the variation in the RI. Many biomolecules have refractive indices between 1.300 and 1.600, while the refractive index of most gases ranges from 1.000 to 1.600, such as NO_x_^37^, SO_x_, and Cox^38^. In addition, since the number of blood illnesses and other forms of cancer continues to rise, there is a pressing need to detect origins and target tissues. For instance, some research has been conducted on the hemoglobin value of blood^39^ and the ability to see certain types of cancer, such as liver, blood, and breast, using an optical method. In this paper, we calculate the influence of the RI change of an analyte when it changes between 1 and 1.6, while the RI of the medium is assumed to be n = 1.33.

**Figure 4:**
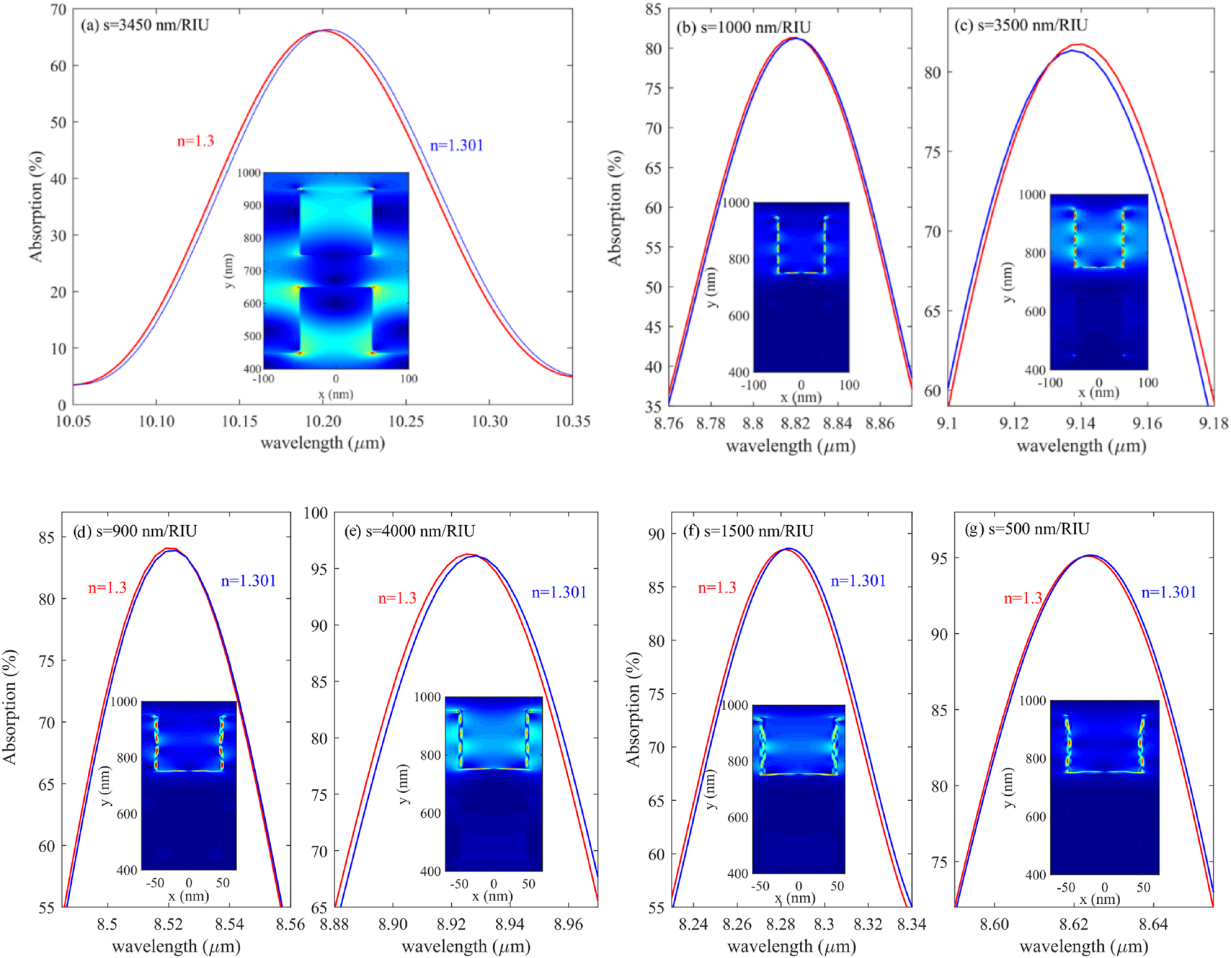
(**a**) structure with only one graphene layer (**b**, **c**) first and second mode of design with two graphene layers (**d**, **e**) first and second mode of structure with three graphene layers (**f**, **g**) first and second mode of system with four graphene layers.

First, we examine the effect of graphene layer number on proposed biosensor S. Figure 4 demonstrates the absorption spectra of the structure with 1,2,3,4 and 5 graphene layers. To analyze the proposed biosensor S, the RI of the analyte has been changed from 1.3 to 1.301, which gives Δn = 0.001 a minimal value representing the accuracy of the proposed structure. In addition, the electric field distributions on the graphene surface have been demonstrated for each case in the inset of Fig. 4a–g. As one can see, the FP pattern can be easily seen from the electric field pattern on the graphene surface and its nodes and antinodes induced by plasmonic wave interference. Each node and antinode crated in electric field distribution on graphene’s surface represents a particular resonance order.

In addition, the S of each case is written in the inset of Fig. 4. For example, the structure of the proposed biosensor with a graphene layer is shown in Fig. 4a. As can be seen, in this case, only one mode exists in the structure absorption profile induced by the FP interferometer and has an S of 3450 nm/RIU. Figure 4b and c show the first and second modes of the proposed biosensor with two layers of graphene, which have an S of 1000 nm/RIU and 3500 nm/RIU, respectively. Figure 4d and e show the response of the structure to the change of the analyte RI as much as 0.0001 for mode 1 and mode 2, respectively, when three layers of graphene are used, as can be seen, mode 1 has a S of 900 nm/RIU and mode 2 has an S of 4000 nm/RIU. Finally, Fig. 4f and g show the optical absorption of the proposed biosensor structure with four layers of graphene/CaF2 sandwich structure, which has an S of 1500 nm/RIU for mode 1 and 500 nm/RIU for mode 2, respectively.

As shown in Fig. 4a, when the structure consists of one layer of graphene, there is only one resonance in 7 µm to 14 µm, but by adding more graphene layers, there are two resonance modes in this range.

**Figure 5:**
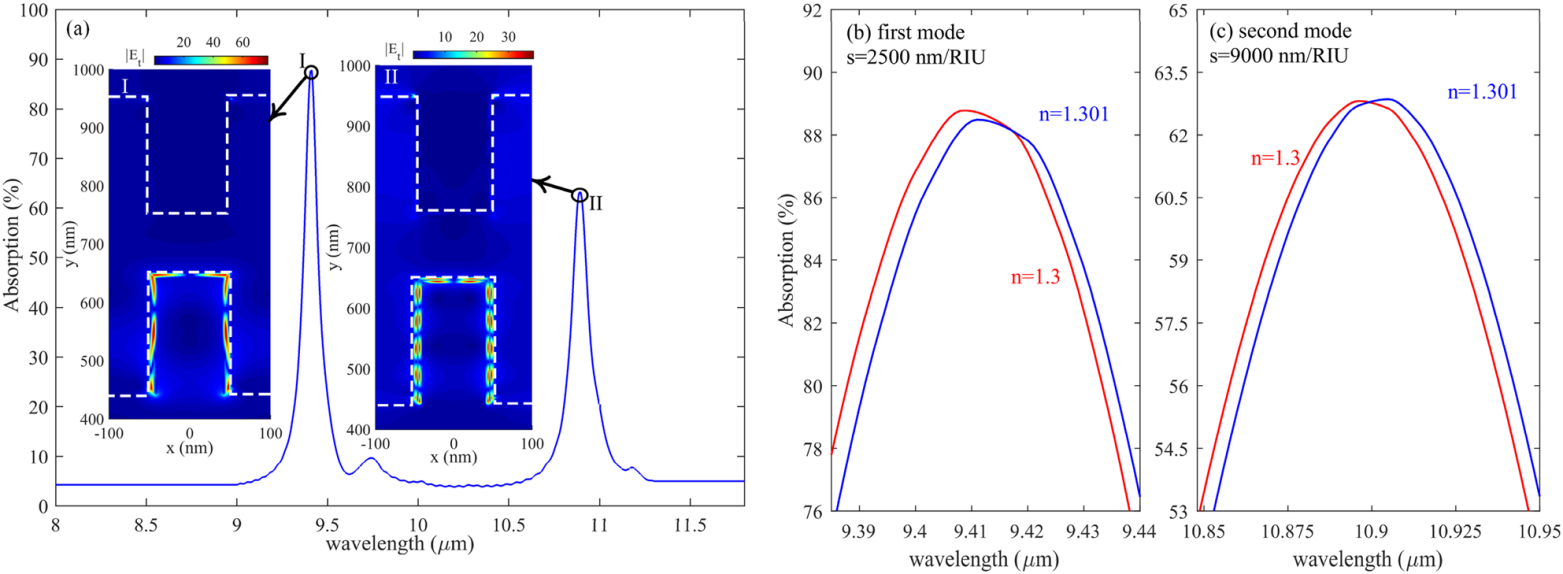
(**a**) Absorption of structure with five graphene layers in the range of 8–12 µm (**b**, **c**) first and the second mode of absorption of the proposed structure with five graphene layers with Δn = 0.001 in the medium index, respectively. The red line represents n = 1.3, and the blue line represents a medium with n = 1.301.

Figure 5 represents the absorption spectra of the proposed biosensor when there is five graphene/Caf_2_ sandwich structure. As shown in Fig. 5a, there are two resonances between 8 µm and 12 µm. By comparing the first resonance, which occurred in 9.4 and its relative electric field distributions, with the second resonance wavelength, which happened at 10.9 µm, it is found that by decreasing the resonance wavelength, the number of FP resonance order which can be defined as the number of nodes and antinodes that appeared in the electric field distribution is increased from k = 6 to k = 12 for resonance wavelength of 9.4 and 10.9 µm, respectively. It is the most well-known behavior of the FP-based structures. Figure 5b,c demonstrates the S of the proposed biosensor structure with five graphene layers.

Comparing Figs. 5 with 4 shows that with five graphene layers, the S is as prominent as 9000 nm/RIU, much more than three, two, and one graphene/Caf_2_ layers. Therefore, we continue our simulation and calculations for a proposed biosensor with five graphene layers after this.

**Figure 6:**
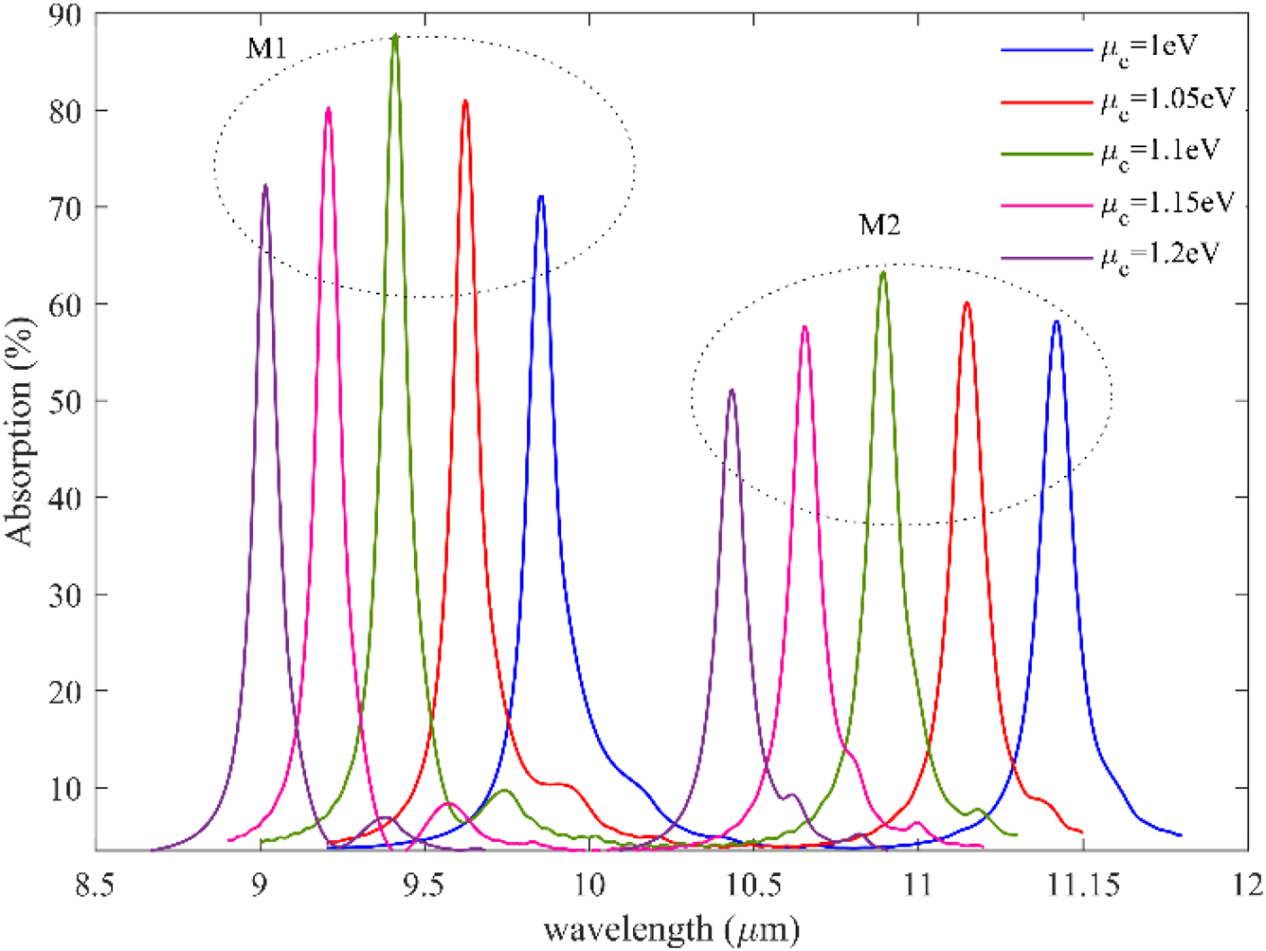
Absorption spectra for two resonance modes and five different fermi levels from 1 to 1.2 with the steps of 0.05 eV, As it is evident from the figure, by increasing the graphene Fermi level, the resonance frequency shifts to high frequencies, which can be translated to resonance wavelength shifts to low wavelength.

We simulated wavelength-dependent absorption for various graphene Fermi levels, as shown in Fig. 6, to demonstrate the adjustable sensing capabilities of graphene plasmonic gratings. The resonant modes illustrate striking blue wavelength variations, which are 9.8 µm to 9 µm for the first mode and 11.14 µm to 10.4 for the second mode when the graphene Fermi level (i.e., chemical potential) varies from 1 to 1.2 eV. Therefore, broadly dynamic tuning operations may be accomplished by electrostatically regulating the Fermi level of graphene. As it is evident from the figure, by increasing the applied gate voltage to the proposed biosensor, the resonance frequency shifts to high frequencies, which can be translated to resonance wavelength shifts to low wavelength, and it can make the proposed structure tunable to the resonance wavelength for both mode and add flexibility to the proposed system. And it adds another advantage to the proposed biosensor.

**Figure 7:**
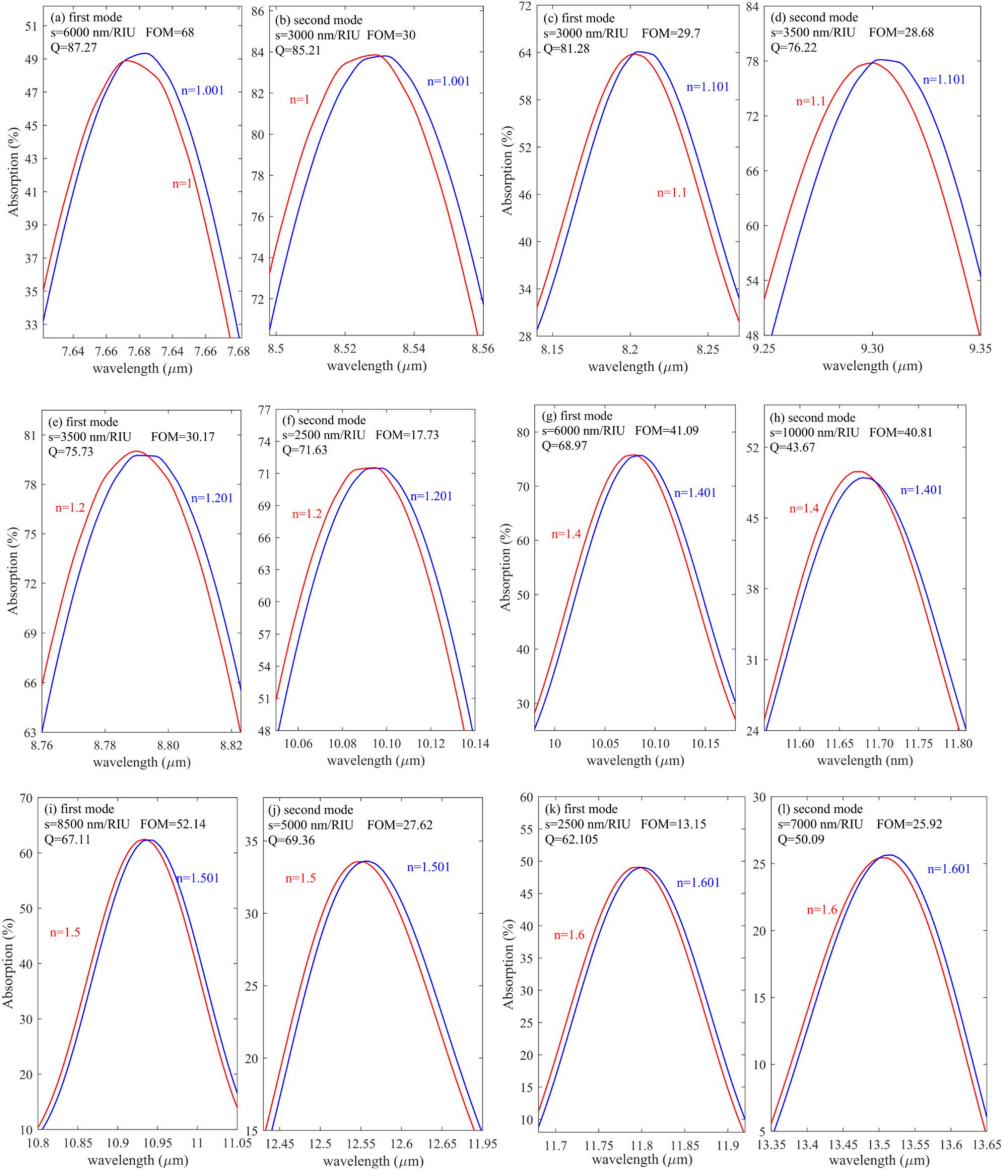
(**a**, **c**, **e**, **g**, **i**, **k**) first mode S and FOM and (**b**, **d**, **f**, **h**, **j**, **l**) second mode S and FOM for n = 1, n = 1.1, n = 1.2, n = 1.4, n = 1.5 and n = 1.6 respectively.

Figure 7a–l represents the shift of resonance wavelengths when the analyte RI changes only 0.001. System's S, FOM and Q are also calculated when RI varies between 1 and 1.6. This range has been studied by considering the large variety of gas alloys and biomolecules’ refractive indices in these around. The outcomes have shown that the suggested structure's FOM changes with sample RI. For instance, the minimum FOM belongs to n = 1.6 with a value of 13.15, and the maximum FOM belongs to n = 1.3 with a value of 81.81. however, its value reaches 68 when the sample RI is 1. CO_2_ is the most crucial gas (n = 1.0002) detected using the proposed sensor. Moreover, some biomolecules, such as MCF-7 (n = 1.401) and MDA-MB-231 (n = 1.399), can be used to diagnose breast cancer, and Jurkat biomolecule (n = 1.390), which is used to detect leukemia, respectively, are in this system identification region^40^ and can be easily detected by the proposed sensor.

As evident from Fig. 7a and b, when the analyte RI changes from 1 to 1.001, the S, FOM, and Q are 6000 nm/RIU 68 and 87.27, respectively, for the first mode. For the second mode shown in Fig. 7b, these values are 3000 nm/RIU, 30, and 85.2, respectively. Moreover, Fig. 7c and d represent the absorption response when the analyte RI varies from 1.1 to 1.101. as it is written in the inset of the figures, the S, FOM, and Q for the first mode are 3000 nm/RIU, 30 and 81.28 respectively in addition as shown in the in Fig. 7 (d) values for the second mode are 3500 nm/RIU, 29, and 76.

Figure 7e and f represent the absorption profile when the analyte RI varies from 1.2 to 1.201. As can be seen, the first and second modes, the S, FOM, and Q, are 3500 nm/RIU, 2500 nm/RIU, and 30, 18, 76, 72, respectively. Figure 7g and h represent the S, FOM, and Q when the RI of the sample changes from 1.4 to 1.401. for the first mode shown in Fig. 7g, the S, FOM, and Q are 6000 nm/RIU, 41, and 69, and for the second mode, 10,000 nm/RIU, 40.8, and 43.67, respectively. Figure 7i and j show the proposed sensor's response when the analyte's RI changes from 1.5 to 1.501 for the first and second modes, respectively. The S FOM and Q are written in the inset of the figures.

Finally, Fig. 7k and l demonstrate the absorption spectra of proposed biosensors for the first and second modes, respectively, when the analyte RI varies from 1.6 to 1.601. the S, FOM, and Q for the first mode were obtained as 2500 nm/RIU, 13.15, and 62.1, respectively, and for the second mode were gained as 7000 nm/RIU, 26, and 50, respectively.

Additionally, the RI discrepancies between liver cancer cells (HCC = 1.347) and normal liver cells (NHCC = 1.345) are from the rank of 0.002, which can be identified using the suggested biosensor^41^. These results have been compared to other SPR-based sense methods, such as fiber optics, which can sense RI with an accuracy of Δn = 0.01 ^42^, gold grating on SiO_2_, which is capable of detecting samples with an accuracy of Δn = 0.005 per RI unit^43^. In contrast, the proposed structure can sense refractive indices with a precision of less than 0.001.

**Figure 8:**
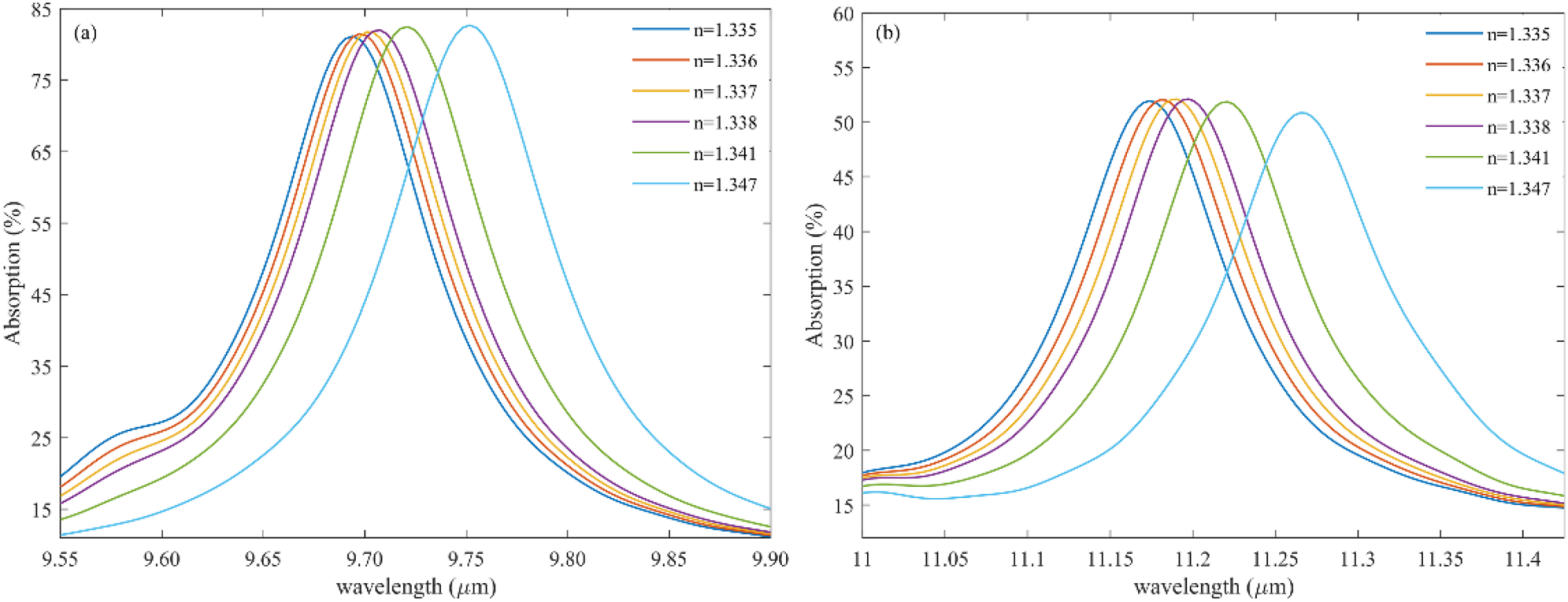
(**a**) M_1_ and (**b**) M_2_ for analyzing the refractive index (RI) of urine.

Different glucose concentrations found in urine samples range in refractive index from 1.332 to 1.340^44^. The refractive index of a patient with diabetes mellitus is relatively high. The mean refractive index of morning urine samples ranges from 1.336 ± 0.0019. In contrast, the refractive index of specific random samples is 1.335 ± 0.0017. As clear from Fig. 8, The higher the glucose concentration (RI = 1.347), the absorption spectrum of the proposed structure shifts to larger wavelengths. Therefore, changing the urine concentration peak shifts according to the sensor's absorption spectrum.

Figure 8 shows the capability of detecting urine concentration with precise accuracy. Figure 8a represents the absorption response of the first mode of the proposed structure for different glucose or urine concentrations whose RI changes from 1.335 to 1.347. likewise, Fig. 8b illustrates the absorption spectra of the proposed biosensor for the second mode when the urine concentration changes in the sample.

By looking precisely at Fig. 8, it can be understood that when the absorption profile peaks in large wavelengths, the sample has the highest value of glucose.

Table 2 indicates the key parameter of the proposed sensor, such as S, Q, and FOM for sensing glucose in the sample where the RI of urine varies from 1.335 to 1.347 for both modes. As can be seen, mode II shows high S compared with mode I. Likewise, the FOM and Q of the proposed structure for sensing glucose for mode II are large. Therefore, in this case, to have a tremendous improvement, choosing the second mode, which happens in large wavelengths, is suitable.

**Table 2:** The S, FOM, and Q for the diabetes sample with different concentration.

Refractive index (RI) of urine	Sensitivity		FWHM		FOM		Q factor	
	Mode 1	Mode 2	Mode 1	Mode 2	Mode 1	Mode 2	Mode 1	Mode 2
1.335	3000	8000	87	100	34.48	80	111.42	111.7
1.336	3000	8500	101	122	29.70	69.67	96	91.64
1.337	4000	8000	116	111	34.48	72.07	83.63	100.85
1.338	6000	7000	124	110	48.38	63.63	78.28	101.81
1.341	4600	8000	116	120	39.65	66.66	83.80	93.54
1.347	5166	7300	105	107	49.2	68.22	92.87	105.37

Diabetes mellitus is a chronic metabolic condition that affects millions of people worldwide. This article examines the response of a graphene grating-based sensor to the refractive index of urine samples (human renal fluids) to diagnose diabetes mellitus. Evanescent field interaction with the high glucose concentration, known as "hyperglycemia" (RI 1.347) sample over the proposed sensor, causes the refractive index to change. The proposed biosensor can also help detect low glucose levels (RI 1.2) and is named "hypoglycemia."

Physically, a urine sample will change the surface tension, gravity, and refractive index due to the presence of glucose. Specific characteristics of label-free biosensing are their resistance to electromagnetic interference, compact design, the potential to integrate into the lab on a chip, etc. these sensors lead to significant developments in diagnosis, food safety management, pharmaceutical development, and monitoring of environmental hazards, and recently, it has become crucial to use a noninvasive glucose sensing method compared to the traditional blood pinpricking method. Also, some cancer such as Adrenal Gland Cancer, Blood Cancer, Breast Cancer I, Breast Cancer II, Cervical Cancer, and skin cancer have been investigated precisely.

**Figure 9:**
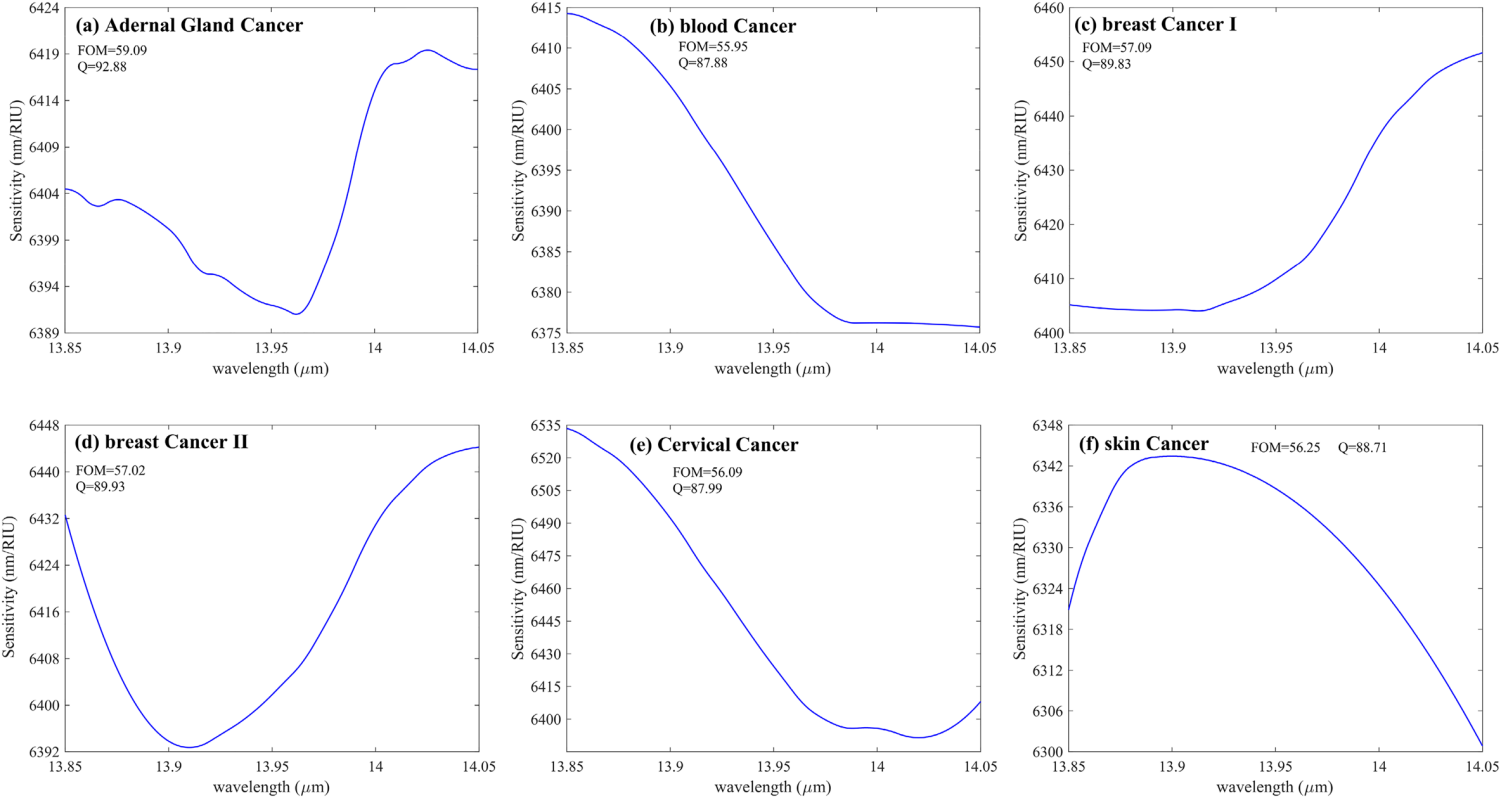
(**a**–**f**) S versus wavelength for six different cancer cells shown in Table 3. The maximum S belongs to (**e**), demonstrating the S of the proposed structure to Cervical Cancer. Other critical parameter variations of the designed sensor for these cancers are negligible, such as Q and FOM inscribed inside the figures.

Figure 9 demonstrates the S of the various cancerous cells shown in the Table 3 Concerning the incident wavelength. The graph mentioned earlier shows that type II breast cancer cells have greater S than other cancer cells. On the other hand, it is undoubtedly evident that the fluctuations of sensitivities concerning the incident source wavelength are negligible.

**Table 3:** The summary of the response of the proposed structure to examine cancers in Fig. 9

Cell (Cancer) Name	Refractive index	Maximum sensitivity (nm/RIU)	FOM	Quality factor	ReferenceS
-12 (Adrenal Gland_Cancer)	1.398	6419	59.09	92.88	^45,46^
Jurkat (Blood_Cancer)	1.390	6415	55.95	87.88	^45,46^
(MDA)-(MB)-231(Breast_Cancer I)	1.399	6450	57.09	89.83	^45,46^
MCF-7 (Breast_Cancer II)	1.401	6444	57.02	89.93	^45,46^
HeLa (Cervical_Cancer)	1.392	6335	56.09	87.99	^45,46^
Basal (Skin_Cancer)	1.380	6342	63.42	88.71	^45,46^

Although the S fluctuation of the proposed structure concerning wavelength variations is insignificant for the diverse types of cancers shown in Table 3, it can be considered constant over the wavelength range shown in Fig. 9 (a)-(f). This independence of the S from the sources' wavelength variations adds another merit to the proposed structure. But as evident in Fig. 9, the maximum S of the system for detecting PC-12 (Adrenal Gland Cancer) reaches about 6420 nm/RIU at the wavelength of 14.05 µm. In addition, the maximum S of Blood Cancer at the wavelength of approximately 13.85 µm goes 6415 nm/RIU. Also, the structure can identify (MDA)-(MB)231(Breast Cancer I) with a maximum S of around 6450 when the incident light wavelength has been fixed to 1.405 µm. For Breast Cancer type II, the thoroughgoing S, according to Fig. 9d, reaches about 6445 nm/RIU at 14.05 µm. Figure 9e represents the S concerning the incident wavelength of Cervical Cancer as it is evident from the figure that the amount of S has been sharply decreased from 6535 nm/RIU at a fixed wavelength of 13.85 *µm* to almost 6400 nm/RIU where the incident wavelength has been fixed to 14.05 µm. Also, the structure can sense Skin Cancer with a maximum S of nearly 6350 nm/RIU at 13.9 µm.

As evident from Fig. 9a–f, the following facts can be extracted from these figures. First of all, it is shown that the S variation concerning the wavelength variation is highly insignificant and negligible. The second circumstance is that the FOM and Q of all Cancers are almost constant. The maximum value of FOM and Q reaches 59 and 92, respectively, for Fig. 9a, which demonstrates Adrenal Gland Cancer, and the minimum value of FOM and Q decreased to almost 56 and nearly 87, respectively, belonging to Fig. 9b representing the S fluctuation of Blood Cancer when the incident wavelength range changes from 13.85 µm to 14.05 µm.

The lists of refractive indices for the various cancer cells employed in this study are shown in Table 3, taken from^45–51^. Moreover, (MDA)-(MB)-231 and MCF-7 are classified as Types I and II of breast cancer, respectively. The S of each cancer can be defined as the shift in the absorption of the proposed structure when the refractive index changes from cancer RI to its normal cell RI. For example, the Jurkat cell or blood cancer has a refractive index of 1.39 with an 80% concentration, while its normal cell refractive index is 1.376^52,53^. Also, the refractive index of Cervical cancer with an 80% concentration is 1.392, as shown in Table 3 and its normal cell refractive index is 1.368 with 30–70% concentration of the mentioned sample^48,54–58^. The refractive index of Adrenal Gland Cancer is 1.395 with an 80% concentration, and its normal cell owns a RI of 1.381 in the 30–70% concentration^59^. Also, Breast cancer I and type II own the normal cells with the RI of 1.385 and 1.387^60^. Finally, the RI of skin cancer (Basal Cell) with an 80% concentration is 1.38, and its normal cell (basal cell) RI with a concentration in the range of (30% to 70%) is 1.36^59–61^.

The capability of our proposed structure is not limited to these; the proposed sensor can identify and sense viruses inside the sample. The RI of different viruses is given in the previous literature^62^. For example, the RI of the human immunodeficiency virus (HIV) is 1.5, which is entirely covered by the proposed structure. Table 4 demonstrates the S as a function of virus type. As shown, five viruses have been examined with the proposed system. Since the size of some viruses is significant, therefore, to detect a virus, simply the proposed structure can be embedded inside a fluidic sample like blood and water. In the obtained results shown in Table 4 , the viruses are modeled as polystyrene particles with RI taken from^63^ inside a fluidic medium with its radius obtained from^62^.

**Table 4:** The S and resonance wavelengths of the proposed biosensor for both mode one and mode two for four viruses sample.

Virus of interest	Refractive index	Sensitivity (nm/RIU)		Resonance wavelength (µm)		ReferenceS
		M_1_	M_2_	λ_R_(M_1_)	λ_R_(M_2_)	
M13 bacteriophage	1.57	4740	8000	8.732	11.2032	^64,65^
HIV-1	1.5	8010	12,000	8.444	10.7307	^66,67^
Herpes simplex virus type 1	1.41	8100	38,000	10.1502	11.7878	^68,69^
Influenza A virus	1.48	9010	12,000	8.36837	10.5986	^69–71^

The S of the proposed multilayer graphene sensor is compared in Table 5 with some of the previously published graphene-based sensors in reputable journals. This Table shows that our optimized multilayer graphene sensor has a highly competitive sensing performance which can open a new door for integrated optical applications. Since most plasmonic-based sensors structure has a metallic shape to induce LPR in a metal–dielectric interface, our proposed sensor, by taking advantage of the semi-metal feature of graphene, can efficiently generate the plasmonic waves on the graphene surface, which finally, due to propagating the plasmonic waves on graphene surface in the opposite direction, the FP interference has happened which is the base mechanism of the sensing in the suggested biosensors.

**Table 5:** Comparison between this work and other previously published literature.

Design	Highest sensitivity (nm/RIU)	References
A label-free graphene-based nanosensor using surface plasmon resonance	333.3	^72^
MoS2–graphene hybrid nanostructures enhanced localized surface plasmon resonance biosensors	360	^73^
Refractive Index Biosensor Using Metamaterial Perfect Absorber Based on Graphene	2500	^74^
Gold Metasurface Array	3500	^75^
Graphene and C-Shaped Metasurface	3846	^8^
Deformed Graphene	4990	^76^
Graphene-based Chiral Metasurface	6600	^77^
photonic crystal fiber based on gold-graphene layers	8600	^78^
Propose multilayer graphene grating biosensor	38,000	This work

## Conclusion

This study developed a high-performing RI sensor for sensing gas alloys, biomolecules, cancers, and viruses using the excitation of propagation SPs waves in multilayer graphene gratings. For better binding between gas alloys or biomolecules and cancers with a label-free SPR sensor to increase the performance of the proposed design, five graphene layers are embedded in the CaF_2_ gratings. The sensing medium is set between two grating structures. Modifying the structure made it possible to get the highest S, 8500 nm/RIU, and the FOM 52.14 for the first mode (mode 1) and the outstanding S, 10,000 nm/RIU, and the FOM 81 for the second mode (mode 2) for diabetes sensing. Our proposed method and structure can open a new door for integrated optical applications. Since most plasmonic-based sensors structure has a metallic shape to induce LPR in a metal–dielectric interface, our proposed sensor, by taking advantage of the semi-metal feature of graphene, can efficiently generate the plasmonic waves on the graphene surface, which finally, due to propagating the plasmonic waves on graphene surface in the opposite direction, the FP interference has happened which is the base mechanism of the sensing in the suggested biosensors. Also, it is evident that using metal on a graphene surface makes the fabrication process difficult and causes second-order effects. Since FP interference is highly sensitive to its medium RI, the proposed design shows significant improvement in S, FOM, and Q. Additionally, research has been done on the potential sensing properties of various gas alloys and biomolecules. The proposed sensor can sense urine concentration with a maximum S of 8500 nm/RIU and cancers with high S in the 6000 nm/RIU range to 7000 nm/RIU. Also, four viruses, such as M13 bacteriophage, HIV type one, Herpes simplex type 1, and influenza, have been investigated, showing Maximum S of 8000, 12,000, 38,000, and 12,000 nm/RIU, respectively.

## Data Availability

The datasets used and analyzed during the current study are available from the corresponding author upon reasonable request.
